# Non-contiguous finished genome sequence of *Prevotella timonensis* type strain 4401737^T^

**DOI:** 10.4056/sigs.5098948

**Published:** 2014-02-20

**Authors:** Véronique Roux, Catherine Robert, Didier Raoult

**Affiliations:** 1 Aix Marseille Université, URMITE, Faculté de médecine, Aix-Marseille Université

**Keywords:** *Prevotella timonensis*, *Bacteroidetes*

## Abstract

*Prevotella timonensis* strain 4401737^T^ is a member of the genus *Prevotella,* which contains anaerobic Gram-negative bacteria. It was isolated from a human breast abscess. In this work, we describe a set of features of this organism, together with the complete genome sequence and annotation. The 3,169,464 bp long genome contains 2,746 protein-coding genes and 56 RNA genes, including 3 or 4 rRNA operons.

## Introduction

*Prevotella timonenis* strain 4401737^T^(CIP 108522^T^= CCUG 50105^T^) is the type strain of *P. timonensis.* This bacterium was isolated from a human breast abscess [1]. The genus *Prevotella* is comprised of anaerobic Gram-negative bacteria. It currently contains 47 members [2]. Recently, many species of the genus *Prevotella* have been isolated from human sources, often associated with the oral cavity [3-8], but also from feces [9], amniotic fluid [10], blood cultures, lung abscess pus, broncho-alveolar lavages [11] and pleural fluids [12].

Here we present a summary classification and a set of features for *P. timonensis,* together with the description of the non-contiguous finished genomic sequencing and annotation.

## Classification and features

The 16S rRNA gene sequence of *P. timonensis* strain 4401737^T^ was compared with sequences deposited in the Genbank database, indicating that the initial taxonomic classification is correct.

**Figure 1:**
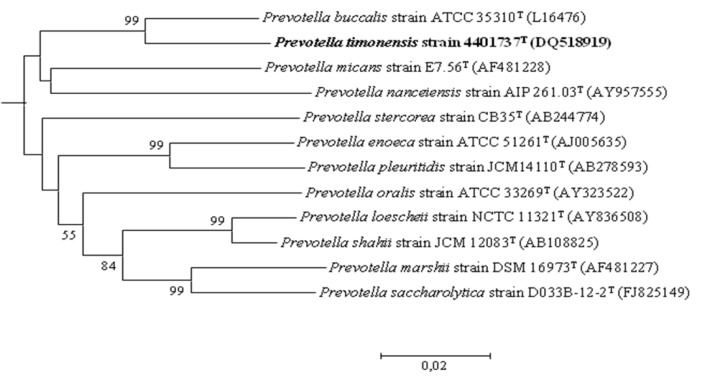
Part of a phylogenetic tree highlighting the position of *Prevotella timonensis* strain 4401737^T^ relative to other type strains within the genus *Prevotella* by comparison of 16S rRNA gene sequences. GenBank accession numbers are indicated in parentheses. Sequences were aligned using CLUSTALX, and phylogenetic inferences obtained using the neighbor joining method within the MEGA 5 software [13]. Numbers at the nodes are percentages of bootstrap values (≥ 50%) obtained by repeating the analysis 1,000 times to generate a majority consensus tree. *Paraprevotella clara* was used as the outgroup (not shown). The scale bar represents 0.002 nucleotide change per nucleotide position.

Figure 1 shows the phylogenetic neighborhood of *P. timonensis* in a 16S rRNA based tree.

The bacterium was first characterized in 2004; it was isolated from a 40-year-old woman who underwent a breast abscess puncture. The organism was in the liquid from the punctured abscess and was cultured in the Timone Hospital microbiology laboratory.

**Figure 2:**
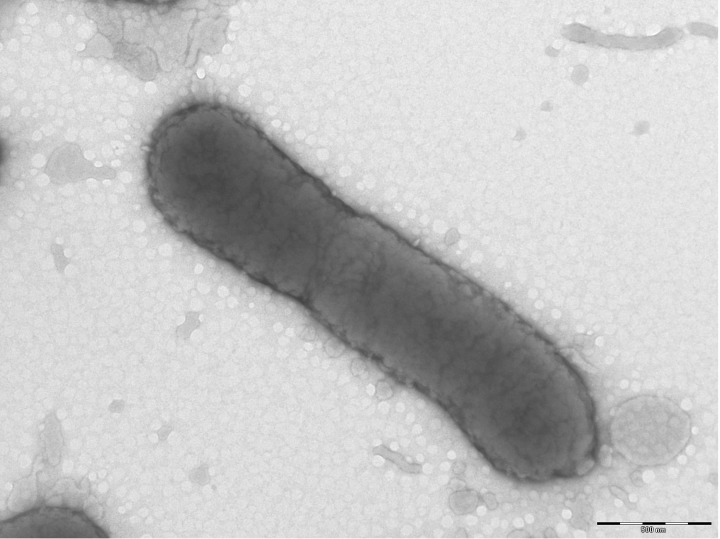
Transmission electron micrograph of *T. timonensis* strain 4401737^T^, using a Morgani 268D (Philips) at an operating voltage of 60kV. The scale bar represents 500 μm.

Cells are rods 0.8-1.4 µm long and 0.3-0.5 µm wide and usually occurred singly. Optimal growth of strain 4401737^T^ occurs at 37°C with a range for growth between 25 and 37 °C. After 72 hours growth on blood sheep agar at 37°C, surface colonies are circular, white-greyish, smooth, shiny, non-pigmented and 1-2 mm in diameter. Carbon sources utilized include ribose, glucose, lactose, maltose and tagatose. Activities of alkaline phosphatase, β-galactosidase, α-glucosidase, N-acetyl-β-glucosaminidase, α fucosidase, arginine arylamidase, leucyl glycine arylamidase, alanine arylamidase are detected. The fatty acid profile is characterized by the predominance of C14:0 (19.5%), C16:0 (15.3%), iso-C14:0 (14%) and a mixture of C18:2 ω6,9c and C18:0 (16%). The size and ultrastructure of cells were determined by negative staining transmission electron microscopy. The rods were 0.8-1.4 μm long and 0.3-0.5 μm wide (Figure 2, Table 1).

**Table 1:** Classification and general features of *Prevotella timonensis* strain 4401737^T^

**MIGS ID**	**Property**	**Term**	**Evidence code^a^**
		Domain *Bacteria*	TAS [14]
		Phylum *Bacteroidetes*	TAS [15,16]
		Class *Bacteroidia*	TAS [15,17]
	Current classification	Order *Bacteroidales*	TAS [15,18]
		Family *Prevotellaceae*	TAS [15,19]
		Genus *Prevotella*	TAS [20-22]
		Species *Prevotella timonensis*	TAS [1]
		Type strain 4401737^T^	TAS [1]
	Gram stain	Negative	TAS [1]
	Cell shape	Rod-shaped	TAS [1]
	Motility	Non motile	TAS [1]
	Sporulation	Non-sporulating	TAS [1]
	Temperature range	Mesophile	TAS [1]
	Optimum temperature	37°C	TAS [1]
MIGS-6.3	Salinity	Not reported	
MIGS-22	Oxygen requirement	Anaerobic	TAS [1]
	Carbon source	Glucose, lactose, maltose, ribose, tagatose	TAS [1]
	Energy source	Chemoorganotroph	NAS
MIGS-6	Habitat	Host	TAS [1]
MIGS-15	Biotic relationship	Free living	TAS [1]
MIGS-14	Pathogenicity Biosafety level Isolation	Unknown 2 Human breast abscess	NAS
MIGS-4	Geographic location	Marseille, France	TAS [1]
MIGS-5	Sample collection time	2004	TAS [1]
MIGS-4.1	Latitude	43°18 N	IDA
MIGS-4.1	Longitude	5°23 E	IDA
MIGS-4.3	Depth	Surface	IDA
MIGS-4.4	Altitude	21 m above sea level	IDA

Evidence codes - IDA: Inferred from Direct Assay; TAS: Traceable Author Statement (i.e., a direct report exists in the literature); NAS: Non-traceable Author Statement (i.e., not directly observed for the living, isolated sample, but based on a generally accepted property for the species, or anecdotal evidence). These evidence codes are from the Gene Ontology project [23]. If the evidence is IDA, then the property was directly observed for a live isolate by one of the authors or an expert mentioned in the acknowledgements.

## Genome sequencing and annotation

### Genome project history

The organism was selected for sequencing on the basis of its phylogenetic position and 16S rDNA similarity to other members of the genus *Prevotella*, and is part of study of the new species characterized in our laboratory. A summary of the project information is shown in Table 2. The EMBL accession number is CBQQ010000001 and consists of 148 contigs (≥ 500 bp) and 25 scaffolds (> 1,500 bp). Table 2 shows the project information and its compliance with MIGS version 2.0 standards.

**Table 2:** Project information

**MIGS ID**	**Property**	**Term**
MIGS-31	Finishing quality	High-quality draft
MIGS-28	Libraries used	One paired end 3-kb library and two Shotgun libraries
MIGS-29	Sequencing platforms	454 GS FLX Titanium
MIGS-31.2	Fold coverage	78.12×
MIGS-30	Assemblers	Newbler version 2.5.3
MIGS-32	Gene calling method	Prodigal
	EMBL ID	CBQQ010000001
	EMBL Date of Release	June 18, 2013
	Project relevance	Study of new species isolated in the URMITE

### Growth conditions and DNA isolation

*P. timonensis* strain 4401737^T^ was grown anaerobically on 5% sheep blood-enriched Columbia agar at 37°C. Five petri dishes were spread and colonies resuspended in 3 ml of TE buffer. Three hundred μl of 10% SDS and 150 μl of proteinase K were then added and incubation was performed over-night at 56°C. The DNA was then extracted using the phenol/chloroform method. The yield and the concentration were measured by the Quant-it Picogreen kit (Invitrogen) on the Genios Tecan fluorometer at 84.3 ng/µl.

### Genome sequencing and assembly

Shotgun and 3-kb paired-end sequencing strategies were performed. A shotgun library was constructed with 500 ng of DNA with the GS Rapid library Prep kit (Roche). For the paired-end sequencing, 5 µg of DNA was mechanically fragmented on a Hydroshear device (Digilab) with an enrichment size at 3-4 kb. The DNA fragmentation was visualized using the 2100 BioAnalyzer (Agilent) on a DNA labchip 7500 with an optimal size of 3.7 kb. The library was constructed according to the 454 GS FLX Titanium paired-end protocol. Circularization and nebulization were performed and generated a pattern with an optimal size of 574 bp. After PCR amplification through 17 cycles followed by double size selection, the single stranded paired-end library was then quantified using the Genios fluorometer (Tecan) at 1070 pg/µL. The library concentration equivalence was calculated as 3.42 x 10^9^ molecules/µL. The library was stored at -20°C until further use. Another shotgun library was constructed with 1μg of DNA as described in the Rapid Library Preparation Method Manual GS FLX+ Series – XL+ except that fragmentation was obtained on Covaris® M220 focused-ultrasonocator^TM^ instead of on a Hydroshear device.

The shotgun and paired-end libraries obtained with the GS-FLX Titanium technology were clonally-amplified with 1 cpb in 4 SV-emPCR reactions, and 0.5 cpb in 2 SV-emPCR reactions with the GS Titanium SV emPCR Kit (Lib-L) v2 (Roche). The yields of the emPCR were 18.7% and 10.9%, respectively, in the 5 to 20% range from the Roche procedure. The shotgun library obtained with the GS-FLX+ technology was clonally-amplified with 3 cpb in 2 SV-emPCR reactions. The yield of the emPCR was 23.95%. Approximately 790,000 beads for the shotgun application and for the 3kb paired end were loaded on the GS Titanium PicoTiterPlate PTP Kit 70x75 and sequenced with the GS FLX Titanium Sequencing Kit XLR70 (Roche). The run was performed overnight and then analyzed on the cluster through the gsRunBrowser and Newbler assembler (Roche). A total of 573,130 passed filter wells were obtained and generated 249.97 Mb with an average length of 424 bp. The passed filter sequences were assembled using Newbler with 90% identity and 40 bp as overlap. The final assembly identified 25 scaffolds and 105 large contigs (>1,500 bp).

### Genome annotation

Open Reading Frames (ORFs) were predicted using Prodigal [24] with default parameters but the predicted ORFs were excluded if they were spanning a sequencing GAP region. The predicted bacterial protein sequences were searched against the GenBank database [25] and the Clusters of Orthologous Groups (COG) databases [26] using BLASTP. The tRNAscan-SE tool [27] was used to find tRNA genes, whereas ribosomal RNAs were found by using RNAmmer [28]. Transmembrane domains and signal peptides were predicted using TMHMM [29] and SignalP [30], respectively. ORFans were identified if their BLASTp *E-*value was lower than 1 x 10^-3^ for alignment length greater than 80 amino acids. If alignment lengths were smaller than 80 amino acids, we used an *E*-value of 1 x 10^-5^. Such parameter thresholds have been used in previous works to define ORFans.

To estimate the mean level of nucleotide sequence similarity at the genome level between *P. timonensis* and *Prevotella* genomes available to date, we compared the only those ORFs only that could be found on the RAST server [31] with a query coverage of ≥60% and a minimum nucleotide length of 100 bp.

## Genome properties

**Figure 3:**
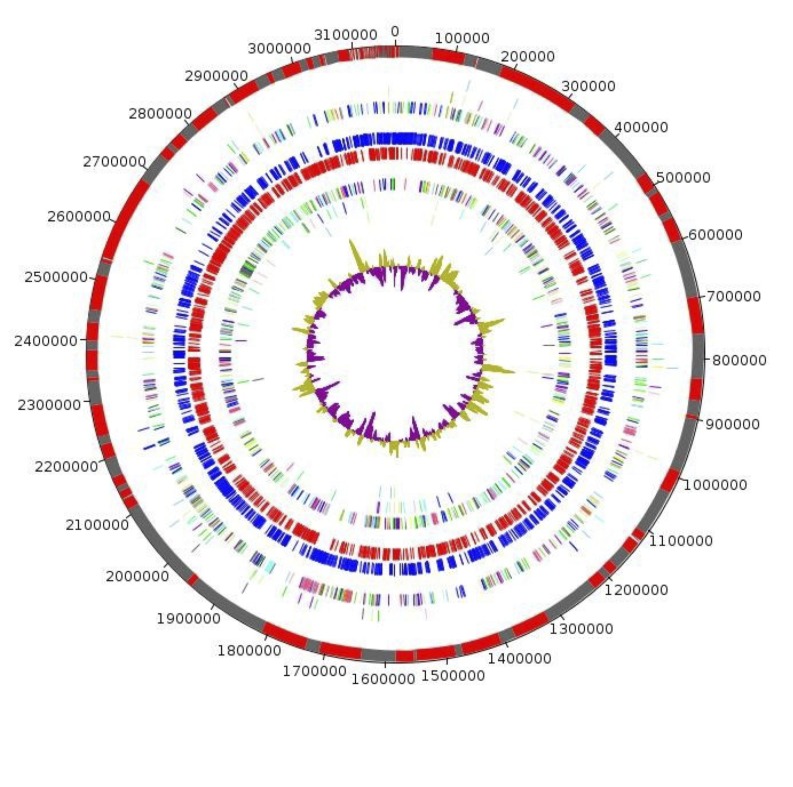
Graphical circular map of *Prevotella timonensis* genome. From outside to the center: Contigs (red / grey), COG category of genes on the forward strand (three circles), genes on forward strand (blue circle), genes on the reverse strand (red circle), COG category on the reverse strand (three circles), GC content.

The genome is 3,169,464 bp long with a 40.50% GC content (Table 3, Figure 3). Of the 2,802 predicted genes, 2,746 were protein-coding genes, and 56 were RNAs. A total of 1,795 genes (65.37%) were assigned a putative function. 198 genes were identified as ORFans (7,21%). The remaining genes were annotated as hypothetical proteins (673 genes (24,51%)). The remaining genes were annotated as either hypothetical proteins or proteins of unknown function. The distribution of genes into COGs functional categories is presented in Table 4. The properties and the statistics of the genome are summarized in Tables 3 and 4.

**Table 3:** Nucleotide content and gene count levels of the genome

Attribute	Value	% of total^a^
Genome size (bp)	3,169,464	100
DNA coding region (bp)	2,758,009	87.02
DNA G+C content (bp)	1,347,151	42.50
Total genes	2,802	100
RNA genes	56	2.00
Protein-coding genes	2,746	98.00
Genes with function prediction	1,795	65.37
Genes assigned to COGs	1,479	53.86
Genes with peptide signals	678	24.69
Genes with transmembrane helices	540	19.66

a) The total is based on either the size of the genome in base pairs or the total number of protein coding genes in the annotated genome.

**Table 4:** Number of genes associated with the 25 general COG functional categories

**Code**	**Value**	**% of total**	**Description**
J	135	4.92	Translation
A	0	0	RNA processing and modification
K	87	3.17	Transcription
L	179	6.52	Replication, recombination and repair
B	0	0	Chromatin structure and dynamics
D	23	0.84	Cell cycle control, mitosis and meiosis
Y	0	0	Nuclear structure
V	44	1.60	Defense mechanisms
T	42	1.53	Signal transduction mechanisms
M	171	6.23	Cell wall/membrane biogenesis
N	2	0.07	Cell motility
Z	0	0	Cytoskeleton
W	0	0	Extracellular structures
U	35	1.27	Intracellular trafficking and secretion
O	73	2.66	Posttranslational modification, protein turnover, chaperones
C	74	2.69	Energy production and conversion
G	107	3.90	Carbohydrate transport and metabolism
E	87	3.17	Amino acid transport and metabolism
F	62	2.26	Nucleotide transport and metabolism
H	62	2.22	Coenzyme transport and metabolism
I	42	1.53	Lipid transport and metabolism
P	98	3.57	Inorganic ion transport and metabolism
Q	12	0.44	Secondary metabolites biosynthesis, transport and catabolism
R	206	7.5	General function prediction only
S	80	2.91	Function unknown
X	1267	46.14	Not in COGs

The total is based on the total number of protein coding genes in the annotated genome.

## Comparison with other *Prevotella* genomes

To date 33 genomes from species belonging to the genus *Prevotella* have been sequenced.

Whole genome sizes ranged between 2.42 Mb (*P. bivia* and *P. amnii*) and 3.62 Mb (*P. ruminicola*). The G+C content of the genomes was was between 36.5% for *P. amnii* and 55.9% for *P. dentalis*. 16S rRNA gene sequence comparison was performed to obtain phylogenetic analysis of *Prevotella* species. A cluster including *P. bergensis*, *P. dentalis*, *P. multisaccharivorax*, *P. buccae*, *P. baroniae*, *P. dentasini*, *P. denticola* and *P. multiformis* was identified. From this group. the genomes of *P. bergensis*, *P. dentalis*, *P. multisaccharivorax*, *P. buccae*, *P. denticola* and *P. multiformis* have been sequenced. It is interesting to note that these genomes showed the highest G+C contents (47.6-55.9%) among the bacteria included in the genus *Prevotella*. A more in-depth study will allow us to determine if this group of bacteria shared an evolutionary path’.

The genome of another strain of the species *P. timonensis* was sequenced, strain CRIS 5C B1. The genome of *P. buccalis,* which is the more closely related species to *P. timonensis* when 16S rRNA encoding gene sequences were compared, has also been sequenced.*P. timonensis* strain 4401737^T^ shared a mean sequence similarity of 96.45% (60.2-100%) with *P. timonensis* strain CRIS 5C B1 and of 84.02% (60-100%) with *P. buccalis*.

The partition of the coding sequences into subsystems [31] is similar for the two genomes except for the transposable elements, whose numbers are significantly higher in strain 4401737^T^.
